# Differential regulation of cytotoxicity pathway discriminating between HIV, HCV mono- and co-infection identified by transcriptome profiling of PBMCs

**DOI:** 10.1186/s12985-014-0236-6

**Published:** 2015-01-27

**Authors:** Jing Qin Wu, Monica Miranda Saksena, Vincent Soriano, Eugenia Vispo, Nitin K Saksena

**Affiliations:** School of Biomedical Sciences and Pharmacy, Faculty of Health, The University of Newcastle, University Drive, Callaghan, Newcastle, NSW 2308 Australia; Herpes Virus Pathogenesis Lab, Center for Virus Research, Westmead Millennium Institute, University of Sydney, Westmead, Sydney, NSW 2145 Australia; Department of Infectious Diseases, Hospital Carlos III, Sinesio Delgado 10, 28029 Madrid, Spain; Retroviral Genetics Division, Center for Virus Research, Westmead Millennium Institute & Westmead Hospital, University of Sydney, Westmead, Sydney, NSW 2145 Australia

**Keywords:** HIV, HCV, HIV/HCV co-infection, Transcriptome, Cytotoxicity pathway

## Abstract

**Background:**

Despite the easy accessibility and diagnostic utility of PBMCs and their potential to show distinct expression patterns associated with the accelerated disease progression in HIV/HCV co-infection, there has not been a systematic study focusing on the global dysregulations of the biological pathways in PBMCs from HIV, HCV mono- and co-infected individuals. This study aimed at identifying the transcriptome distinctions of PBMCs between these patient groups.

**Methods:**

Genome-wide transcriptomes of PBMCs from 10 HIV/HCV co-infected patients, 7 HIV+ patients, 5 HCV+ patients, and 5 HIV/HCV sero-negative healthy controls were analyzed using Illumina microarray. Pairwise comparisons were performed to identify differentially expressed genes (DEGs), followed by gene set enrichment analysis (GSEA) to detect the global dysregulations of the biological pathways between HIV, HCV mono- and co-infection.

**Results:**

Forty-one, 262, and 44 DEGs with fold change > 1.5 and FDR (false discovery rate) <0.05 for the comparisons of HCV versus co-infection, HIV versus co-infection, and HIV versus HCV were identified, respectively. Significantly altered pathways (FDR < 0.05), featured by those involved in immune system, signaling transduction, and cell cycle, were detected. Notably, the differential regulation of cytotoxicity pathway discriminated between HIV, HCV mono- and co-infection (up-regulated in the former versus the latter group: co-infection versus HIV or HCV, HIV versus HCV; FDR <0.001 ~ 0.019). Conversely, the cytokine-cytokine receptor interaction pathway was down-regulated in co-infection versus either HCV (FDR = 0.003) or HIV (FDR = 0.028). For the comparison of HIV versus HCV, the cell cycle (FDR = 0.016) and WNT signaling (FDR = 0.006) pathways were up- and down-regulated in HIV, respectively.

**Conclusions:**

Our study is the first to identify the differential regulation of cytotoxicity pathway discriminating between HIV, HCV mono- and co-infection, which may reflect the distinct patterns of virus-host cell interactions underlying disease progression. Further inspection of cytotoxicity pathway has pinned down to the expression of the KIR genes to be associated with specific patterns of particular virus-host interactions. Between HIV and HCV, the altered cell cycle and WNT signaling pathways may suggest the different impact of HIV and HCV on cell proliferation and differentiation.

**Electronic supplementary material:**

The online version of this article (doi:10.1186/s12985-014-0236-6) contains supplementary material, which is available to authorized users.

## Introduction

Infection by HIV is commonly complicated by co-infection with hepatitis C virus (HCV) due to shared routes of transmission. Overall, about 30% of HIV-infected individuals are co-infected with HCV in the United States and Europe [1]. While AIDS-associated mortality has declined substantially [2] following the release of highly active antiretroviral therapy (HAART), HCV-related liver disease has become the leading cause of death in HIV-infected individuals [2,3].

In co-infected individuals, HIV worsens the course of HCV infection and *vice versa*. Liver fibrosis progresses more rapidly in those patients than in mono-infected ones, resulting in increased rates of cirrhosis and complications such as decompensation and hepatocellular carcinoma (HCC) [4-7]. Compared to HCV mono-infected patients, co-infected individuals are also less likely to achieve viral clearance, and have higher HCV viral loads and more frequent HCV relapses after anti-HCV therapy [8-11]. Similarly, the possible role of HCV to accelerate HIV disease progression has also been reported by previous studies, which have shown the impaired CD4+ T cell count recovery during HAART, higher risk of AIDS, and liver disease-related deaths in co-infected individuals in comparison to HIV mono-infected ones [12-19]. Moreover, adverse drug reactions to HAART such as rash and hepatoxicity in HIV patients are exacerbated in co-infected patients, making the virological control of HIV harder to achieve [20-24].

Despite the clinical significance of HIV/HCV co-infection, the mechanisms underlying the accelerated progression of the diseases in the co-infected patients remain elusive. Using techniques such as real-time PCR and microarray, gene expression profiling has provided a unique opportunity for understanding virus-host interactions at the transcriptional level. Research by PCR and multiplex assays has revealed that in co-infected versus mono-infected individuals, the former had decreased levels of inflammatory cytokines (IL4, IL8, IL10, IL12, TNF-α, and IFN-γ), increased levels of TGF-β [25,26], impaired response to IFN-α [27], and the association of IFN-γ production with CD4+ T cell counts [25,28]. While PCR studies mainly focused on cytokines, microarray analyses enabled a genome-wide view of transcriptome dysregulations by showing a range of important biological themes associated with the co-infection, including aberrant immune activation, regulation, and differentiation, impaired innate immunity, and dysfunctions of NK and dendritic cells in liver biopsies and PBMCs from co-infected patients [29,30]. Recently, two studies have also demonstrated the dysregulation of cell cycle and metabolism in T cell subsets from co-infected individuals [31,32].

In contrast, studies on the transcriptome of peripheral blood mononuclear cells (PBMCs) from HIV/HCV co-infection are limited despite their easy accessibility and diagnostic utility in clinical settings. To date, only two studies have focused on transcriptomes of PBMCs from HIV, HCV mono- and co-infected individuals [29,30]. While the study by Rasmussen *et al.* has compared the transcriptomes of PBMCs only between HCV infected versus HCV and HIV co-infected individuals, the analysis by Kottilil *et al.* has centered on gene clusters based on differentially expressed genes (DEGs). However, neither of them has looked into the global dysregulations of the biological pathways in PBMCs between HIV, HCV mono- and co-infected individuals. In view of the easy accessibility and diagnostic utility of PBMCs and their potential to show distinct expression patterns associated with the HIV and/or HCV-host cell interactions underlying disease progression, this research studied the transcriptomes of PBMCs from HIV+ patients (HIV; n = 7), HCV+ patients (HCV; n = 5) and HIV/HCV co-infected patients (HH; n = 10) along with HIV/HCV sero-negative healthy controls (CTR; n = 5; Additional file 1). Focusing on significantly altered KEGG pathways detected by gene set enrichment analysis (GSEA), our analysis has shown for the first time, the significant alterations of cytotoxicity pathway differentiating between HIV, HCV mono- and co-infection in PBMC transcriptome profiling. In HH versus HIV or HCV, the down-regulation of cytokine-cytokine receptor interaction and up-regulation of metabolic pathways clearly indicated aberrant immune activation and more severely perturbed metabolism in co-infection. Between HIV and HCV mono-infection, the cell cycle and WNT pathways were differentially regulated, which may implicate the distinct impact of HIV and HCV on cell proliferation.

## Results

### Identification of differentially expressed genes

**Figure 1:**
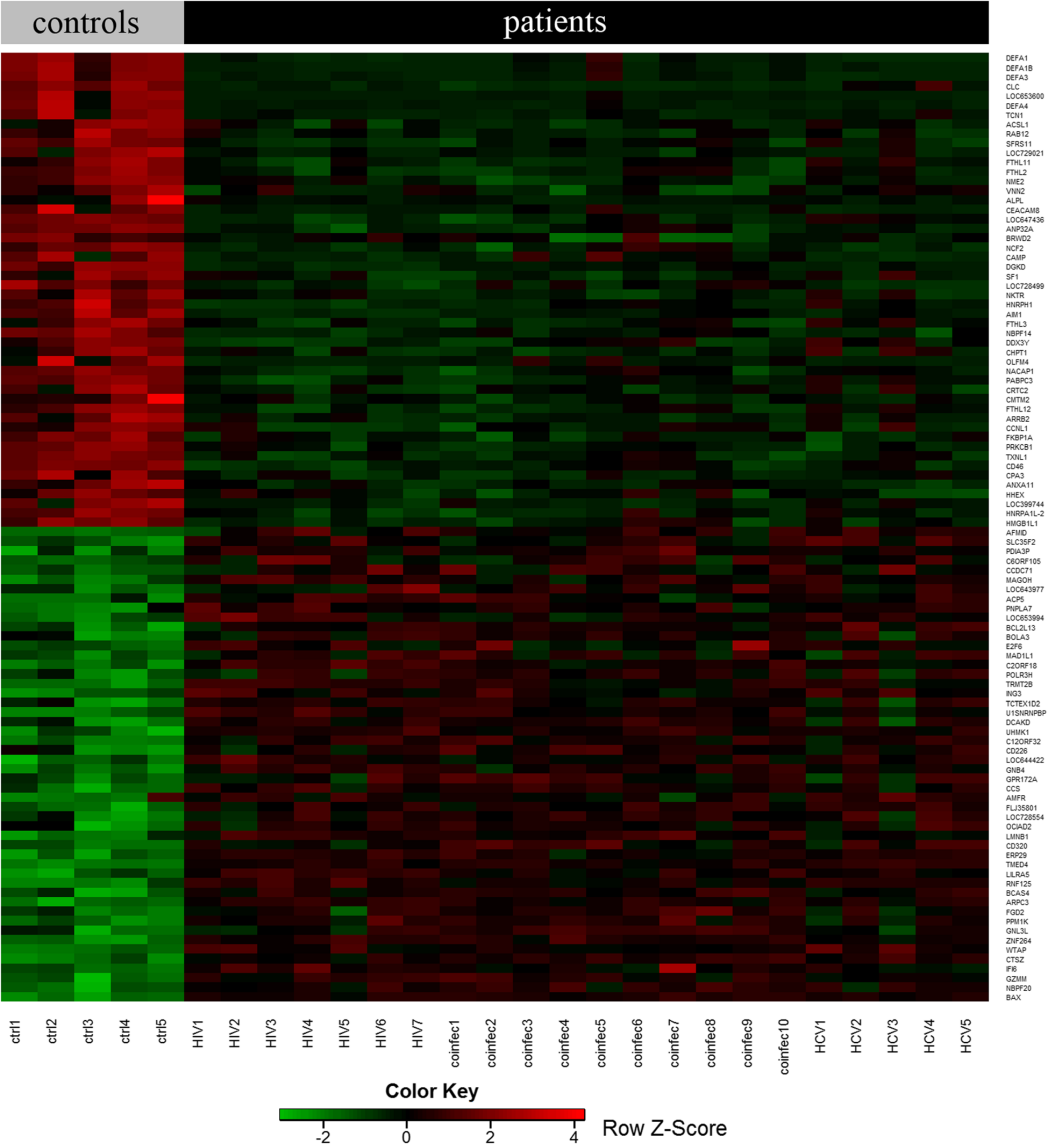
**Heatmap for the differentially expressed genes separating controls from infected patients.** Each row represents a gene and each column represents a sample. The top 50 down- and up-regulated genes (FDR < 0.05; Additional file 2) common in HH, HIV, and HCV versus healthy controls are shown. Red indicates higher expression and green indicates lower expression. Controls: ctrl1-5; HIV+ patients: HIV1-7; HIV/HCV co-infected patients: coinfec1-10; HCV+ patients: HCV1-5.

Genome-wide transcriptomes of PBMCs from four study groups including HIV, HCV mono- and co-infection along with healthy controls (referred to as HIV, HCV, HH, and CTR, respectively; Additional file 1) were analyzed using Illumina microarray. Each patient group was compared to the healthy controls and 2605, 2839, and 2260 differentially expressed genes (DEGs) with False Discovery Rate < 0.05 (FDR < 0.05; FDR is a widely used statistical method for multiple test correction) and fold change >1.5 in HH, HIV, and HCV were detected, respectively (Additional file 2). The DEGs present across the comparisons clearly segregated all the patients from controls as demonstrated by the heatmap (Figure 1). As our major goal was to investigate the transcriptome distinctions between mono- and co-infected groups, our subsequent analysis focused on direct comparisons between them.

For the pairwise comparisons of HCV versus HH, HIV versus HCV, and HIV versus HH, 41, 44, and 262 DEGs, respectively, with FDR < 0.05 and fold change >1.5 were identified (Table 1; Additional file 3). The functional classification of the DEGs based on the biological process from gene ontology (GO) revealed that developmental, immune system, and metabolic processes were the major biological themes across these DEG lists. For example, between HCV and HH, 15 out of 41 DEGs played important roles in development and metabolism (WNT7A, CDKN1A, CDKN1B, DUSP2, EGR2, FOS, ISYNA1, MCM2, NR4A1, PAK1IP1, PRKACG, PTGS2, RGS2, SOX30, and XRN2) and 3 DEGs were directly involved in the immune system (CD28, OSM, and CD40LG). Between HIV and HCV, 20 out of 44 DEGs were involved in development and metabolism (ADAP2, FOXC1, HIPK2, HMGB2, ISYNA1, KDSR, KIF5C, MCM2, MRPL39, ODC1, PDE3B, PLS1, PRKACG, QTRTD1, RPS6KA3, SLC35A2, SOX30, TATDN3, TERF1, and ZNF507). Between HIV and HH, 12 DEGs were involved in the immune system (CAMLG, CD99L2, CLIC4, CNIH, FPR3, HIATL1, HLA-DPB1, IKBKB, IL6, KLF10, NLRP3, and OSM), and 102 out of 262 DEGs were engaged in development and metabolism. These 102 DEGs included critical genes in cell cycle (e.g. CDKN1A and CDKN2C), genes encoding for transcription factors (e.g. NFATC3 and FOXC1), growth factors (e.g. EGR2), and solute carriers (e.g. SLC25A26).

**Table 1:** **Number of differentially expressed genes (DEGs; fold change > 1.5 and FDR < 0.05) and differentially regulated KEGG pathways (FDR < 0.05) derived from pairwise comparisons between patients groups**

		**HIVvsHH**	**HCVvsHH**	**HIVvsHCV**
**DEGs**	**Up-regulated**	188	35	18
	**Down-regulated**	74	6	26
	**Total**	262	41	44
**KEGG pathways**	**Up-regulated**	13	6	17
	**Down-regulated**	12	21	1
	**Total**	25	27	18

Up-regulated and down-regulated in the former group; vs: versus.

### Pathway analysis by GSEA

The GSEA revealed that for the pairwise comparisons of HIV versus HH, HIV versus HCV, and HCV versus HH, 25, 18, and 27 pathways were significantly regulated, respectively (FDR < 0.05; Table 1; Additional file 4). The significant pathways detected in each comparison could all be divided into five biological themes, including (1) immune system; (2) signaling transduction and cell cycle; (3) metabolism; (4) genetic information processing; and (5) human diseases.

For the immune system, the innate immune related pathways including Toll signaling, IgA production, antigen processing and presentation pathways as well as cytokine-cytokine receptor interaction, were significantly altered in 2 of the 3 pairwise comparisons (Additional file 4). Moreover, natural killer (NK) cell mediated cytotoxicity pathway was significantly altered in all the comparisons (up-regulated in HH versus HIV or HCV and in HIV versus HCV, FDR from <0.001 to 0.019; Table 2). For the signaling transduction and cell cycle, notable was the significant up-regulation of p53 pathway in HIV versus HH (FDR = 0.029) as well as the dysregulation of WNT signaling (up-regulated in HCV, FDR = 0.006) and cell cycle (down-regulated in HCV, FDR = 0.016) pathways between HIV and HCV.

**Table 2:** **Core genes contributing to the differential regulation of the critical pathways between HIV, HCV mono- and co-infection identified by GSEA**

**Pathway**	**Comparison**	**Direction**	**Size**	**NOM p-val**	**FDR**	**Core enrichment genes**
KEGG_NATURAL_KILLER_CELL_MEDIATED_CYTOTOXICITY	HIVvsHH	HH-up	134	0.000	0.018	HLA-G, SHC1, TNFSF10, VAV1, MAP2K2, RAC2, PAK1, KLRD1, VAV3, KLRC2, LCK, PLCG1, PIK3CD, KIR2DS1, PTPN6, PPP3CB, SH2D1B, GZMB, FYN, ICAM2, ITGAL, NCR3, KIR2DL3, ZAP70, MAPK1, PTK2B, PRKCA, NFATC3, KIR2DL4, IFNAR1, PPP3CA, KIR3DL2, KIR2DS5, KIR2DL5A, KIR3DL1, KIR2DL1, PRF1
KEGG_NATURAL_KILLER_CELL_MEDIATED_CYTOTOXICITY	HCVvsHH	HH-up	134	0.000	0.000	ZAP70, PLCG1, SHC3, ICAM2, PIK3R2, HCST, SH2D1A, KLRC1, SH2D1B, KIR2DS4, KIR2DS1, NCR3, KLRC2, KIR2DS3, PTK2B, NCR1, NFATC3, ITGAL, MAPK1, TNFSF10, PTPN6, FASLG, IFNG, CD244, KIR2DL4, IFNAR1, KIR2DS5, KIR2DL3, HLA-C, VAV3, KLRD1, KLRC3, GZMB, KIR3DL1, KIR3DL2, KIR2DL5A, PRF1, KIR2DL1
KEGG_NATURAL_KILLER_CELL_MEDIATED_CYTOTOXICITY	HIVvsHCV	HIV-up	134	0.000	0.019	IFNG, HLA-C, KLRC3, IFNB1, TNF, KLRD1, CD244, FASLG, GZMB, VAV3, SH3BP2, SH2D1A, NCR1, CASP3, GRB2, KIR2DS4, HRAS, TNFSF10, KIR3DL2, FAS, HCST, KIR2DL5A, PIK3R2, KRAS, IFNGR2, KIR2DL3, VAV2, SOS2, BID, ULBP1, KIR2DL1, KIR2DS3
KEGG_CYTOKINE_CYTOKINE_RECEPTOR_INTERACTION	HIVvsHH	HIV-up	262	0.000	0.028	IL6, OSM, CCL3L1, CCL3, IL1B, CCL2, IL8, CXCL2, CCL3L3, TNF, TNFSF9, IFNG, IL1A, CCL20, CCL4L1, CXCL16, CLCF1, IFNB1, VEGFA, CCL28, CCL7, CCL4L2, IL12A, CXCL1, IL23A, CD70, CXCR3, TNFRSF21, TNFRSF9, IL28RA, CD40, TNFRSF13C, IL21R, IFNGR1, TNFRSF4, CD40LG, IL12RB1, CXCR4, IL15, IFNGR2, LTA, EPOR, CXCL9, FAS, CXCR5, IL10RA, FLT4, PRLR, CCL8
KEGG_CYTOKINE_CYTOKINE_RECEPTOR_INTERACTION	HCVvsHH	HCV-up	262	0.000	0.003	OSM, IL6, CXCL2, CCL20, IL1B, IL8, CD40LG, IL1A, VEGFA, CCL3, CCL3L1, CCR4, CXCL1, CXCR5, IL23A, CCL3L3, CXCL16, CXCR4, TNFSF9, TNFRSF4, PRLR, TNFRSF13C, KIT, CXCR3, CXCL6, XCR1, CXCR6, LTA, IL10RA, IFNK, CX3CL1, IL1R2, TNFRSF25, IFNGR1, CLCF1, TNFRSF10A, TNF, CCL28, IL2RA, IL28A, TGFBR2, IL6ST, TGFB3, TNFSF13, FLT3LG, IFNA10, TNFRSF21, CD70, PDGFRA, IL24, CXCL13, TSLP, TNFRSF10C, TNFRSF9, PDGFB, TGFB2, IFNA4, TNFSF4, CCR3, XCL2, EDA, IFNA8, IL7R, IL13RA1, IL4R, CCL7, IFNAR2, TNFSF12
KEGG_CELL_CYCLE	HIVvsHCV	HIV-up	115	0.000	0.016	PTTG1, MCM2, GADD45G, TFDP2, CDC20, CCNE2, CDC16, CDK7, CCNB2, FZR1, MCM4, CDK6, E2F5, WEE1, CCNE1, ANAPC10, TTK, CDC23, ANAPC4, E2F3, MDM2, CDKN2C, BUB1, CDKN2A, CCND1, MAD2L1, ANAPC11, MCM7, YWHAQ, CDC27, CHEK1, TGFB1, GADD45B
KEGG_WNT_SIGNALING_PATHWAY	HIVvsHCV	HCV-up	148	0.000	0.006	WNT5B, NFAT5, CSNK1A1L, CTNNBIP1, CSNK2A2, CUL1, CAMK2A, PLCB1, WNT10A, FZD7, PPP3R1, EP300, PPP3CB, NLK, FOSL1, LRP5, FBXW11, SMAD3, PPP2R1A, DAAM1, TBL1XR1, CAMK2G, DVL2, PLCB2, JUN, PPP3CA, PRICKLE2, SIAH1, TCF7, FZD2, LEF1, AXIN2, PPARD, PRKCA, PRKACG, WNT7A
KEGG_P53_SIGNALING_PATHWAY	HIVvsHH	HIV-up	67	0.004	0.029	SESN3, CDKN1A, GADD45A, THBS1, PMAIP1, CCNG2, SESN1, CASP9, CCNE1, CCND1, GADD45B, CASP8, CHEK1, CCNE2, CCNB2, FAS, MDM2, MDM4, BID, TP53I3, EI24, CDKN2A, SESN2, RCHY1, SERPINE1, CD82
KEGG_RENAL_CELL_CARCINOMA	HCVvsHH	HCV-up	70	0.000	0.036	JUN, VEGFA, HIF1A, EGLN1, GRB2, RAF1, EPAS1, SLC2A1, TGFB3, PIK3CA, NRAS, MAPK3, RAPGEF1, CUL2, PDGFB, TGFB2, KRAS, CREBBP

Size: Gene set size (number of genes in a particular gene set); NOM p-val: nominal p value; FDR: false discovery rate.

For the metabolism, a range of pathways were found to be down-regulated for either HIV or HCV versus HH, covering the metabolism of carbohydrate, lipid, amino acid, and nucleotide (Additional file 4). For genetic information processing, pathways involved in DNA recombination and repair, tRNA biosynthesis, and proteasome were all identified in the comparison of HCV versus HH, and sporadically in other comparisons. For human diseases, the majority of the pathways across the comparisons were related to infection and/or immune system dysfunction, and the renal cell carcinoma pathway was significantly altered in the comparison of HCV versus HH (FDR = 0.036). The aforementioned pathways with high relevance of HIV, HCV mono- and co-infection diseases were then under further inspection.

### Differential regulation of cytotoxicity pathway across 3 pairwise comparisons of patients

**Figure 2:**
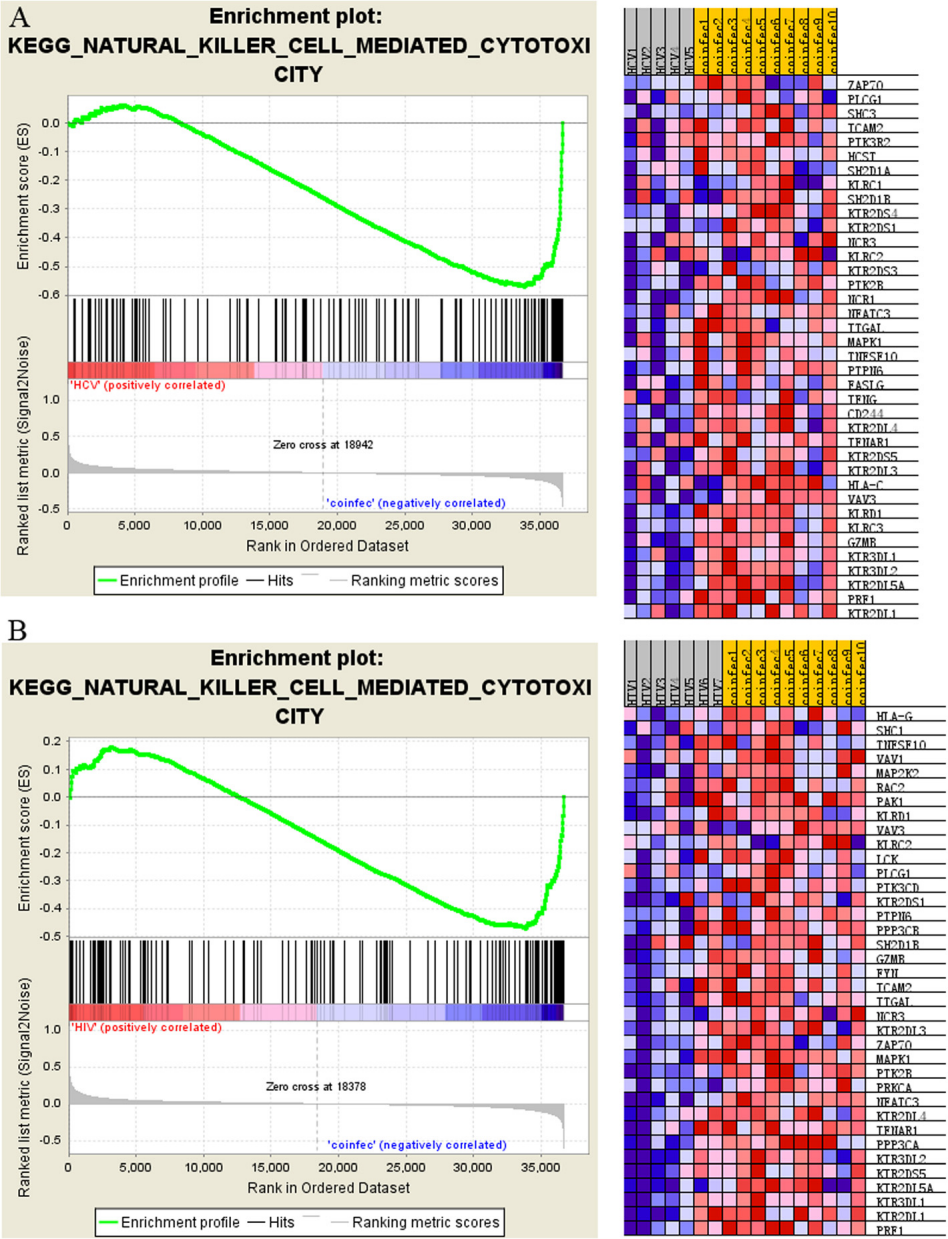
**Enrichment plot and heatmap for NK cell mediated cytotoxicity pathway detected by GSEA. A.** Enrichment plot for the comparison of HCV versus HH and the corresponding heat map of the core enrichment genes. Enrichment plot: Bottom, plot of the ranked list of all genes. Y axis, value of the ranking metric; X axis, the rank for all genes. Genes whose expression levels are most closely associated with the HCV or HH group get the highest metric scores with positive or negative sign, and are located at the left or right edge of the list. Middle, the location of genes from the cytotoxicity pathway within the ranked list. Top, the running enrichment score for the gene set as the analysis walks along the ranked list. The score at the peak of the plot is the enrichment score (ES) for this gene set and those genes appear before or at the peak are defined as core enrichment genes for this gene set. Heat map: The genes that contribute most to the ES, i.e., genes that appear in the ranked list before or at the peak point of ES, are defined as core enrichment genes. Rows, genes; columns, samples. Range of colors (red to blue) shows the range of expression values (high to low). **B.** Enrichment plot for the comparison of HIV versus HH and the corresponding heat map of the core enrichment genes.

**Figure 3:**
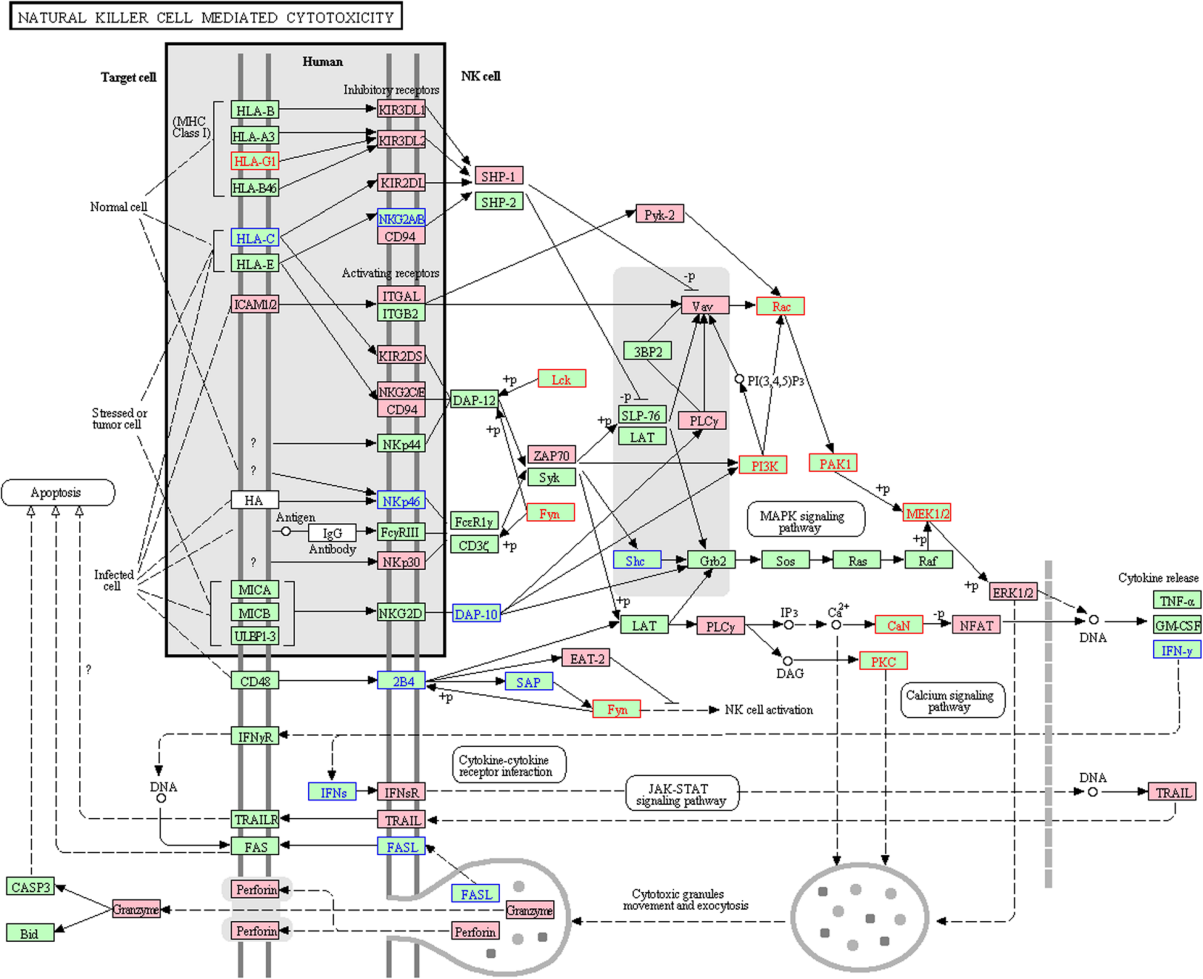
**Coordinately up-regulated genes of NK cell mediated cytotoxicity pathway in HH versus either HCV or HIV.** The pathway figure is adapted from Kyoto Encyclopedia of Genes and Genomes (KEGG; http://www.genome.jp/kegg/). The red and blue front colors highlight the proteins encoded by the coordinately up-regulated genes in HH versus HIV and HCV, respectively. The background color filled with pink highlights the proteins encoded by the commonly up-regulated genes found in both comparisons (HH versus HIV and HH versus HCV).

The NK cell mediated cytotoxicity pathway was up-regulated in HH versus either HIV (FDR = 0.018) or HCV (FDR < 0.001), and in HIV versus HCV (FDR = 0.019). In the comparisons of HH versus HIV or HCV, 37 and 38 out of 134 gene members of this pathway were core enrichment genes, respectively, as shown in the enrichment plots and the heatmaps (Table 2; Figure 2). Between these 2 sets of core genes, 25 were present in both comparisons (Figure 3), which included 15 genes encoding for cell receptors (inhibitory receptors: KIR2DL1, KIR2DL3, KIR2DL4, KIR2DL5A, KIR3DL1, and KIR3DL2; activating receptors: KIR2DS1, KIR2DS5, KLRC2-CD94/NKG2C-KLRD1, NCR3/NKp30, ITGAL/CD11a and its ligand ICAM2; interferon receptor 1 IFNAR1; and TNF-related apoptosis-inducing ligand TNFSF10/TRAIL), 8 genes involved in signaling cascade (kinases: PTK2B/Pyk-2, ZAP70, MAPK1/ERK2 and phosphatase PTPN6/SHP-1; PLC1/PLCγ mediating the production of DAG and IP3; guanine nucleotide exchange factor VAV3; SH2 domain containing 1B SH2D1B/EAT-2; and transcriptional factor NFATC3), and 2 genes encoding for effectors (PRF1/perforin and GZMB/granzyme). The significant up-regulation of cytotoxicity pathway in HIV versus HCV was contributed by 32 core genes, also covering the genes encoding for cell receptors, signaling cascade, and effectors (Table 2). Across all the 3 comparisons, 8 core genes were common (KIR2DL3, KIR2DL5A, KIR2DL1, KIR3DL2, KLRD1, TNFSF10, VAV3, and GZMB).

### Down-regulation of cytokine-cytokine receptor pathway in HIV/HCV co-infection

**Figure 4:**
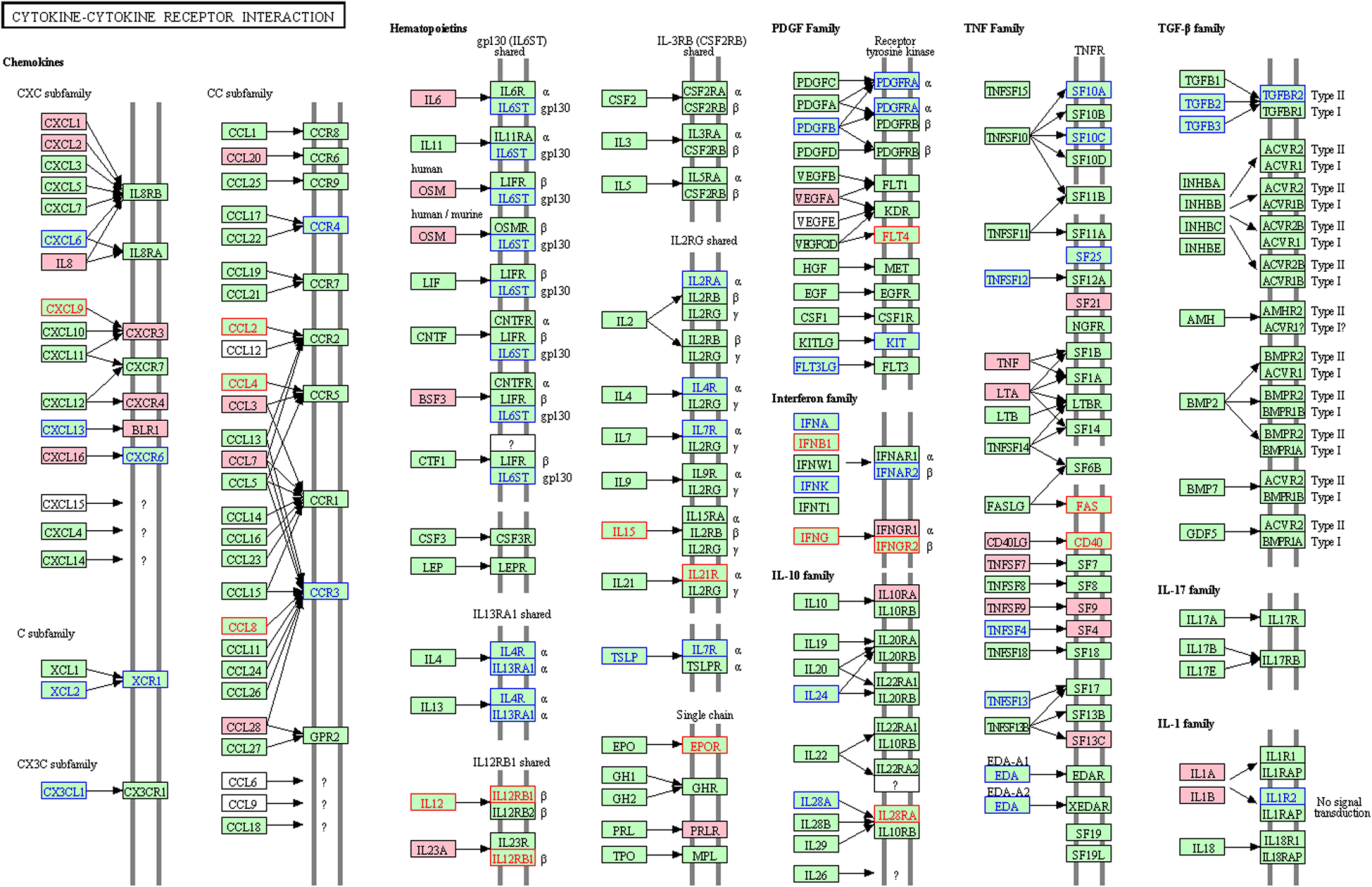
**Coordinately down-regulated genes of cytokine-cytokine receptor pathway in HH versus either HIV or HCV.** The pathway figure is adapted from Kyoto Encyclopedia of Genes and Genomes (KEGG; http://www.genome.jp/kegg/). The red and blue front colors highlight the proteins encoded by the coordinately down-regulated genes in HH versus HIV and HCV, respectively. The background color filled with pink highlights the proteins encoded by the commonly down-regulated genes found in both comparisons (HH versus HIV and HH versus HCV).

The cytokine-cytokine receptor interaction pathway was significantly down-regulated in HH versus either HCV (FDR = 0.003) or HIV (FDR = 0.028; Table 2; Figure 4). Sixty-eight and 49 out of 262 gene members of this pathway were coordinately down-regulated in co-infection versus HCV and HIV, respectively. Figure 4 shows both the unique and common (n = 32) core genes derived from these comparisons, which covered almost every cytokine receptor family (e.g. G protein-coupled receptors including CXC, C, CX3C, and CC subfamilies and interferon family).

### Significant alteration of cell cycle and WNT pathways between HIV and HCV

The cell cycle (FDR = 0.016) and WNT signaling (FDR = 0.006) pathways were significantly up- and down-regulated in HIV versus HCV (Table 2), respectively. Thirty-three out of 115 gene members of cell cycle pathway were coordinately up-regulated in HIV (Table 2; Additional file 5), which appeared to promote G1/S transition and induce arrest in G2/M transition. Twelve genes were associated with the promotion of G1/S transition (cyclin and cyclin dependent kinase: CCND1, CCNE1, CCNE2, CDK6, CDK7; transcription factors and the associated partners: E2F3, E2F5, TFDP2; and mini-chromosome maintenance (MCM) complex: MCM2, 4, 7), whereas only 2 genes were inhibitory (CDK inhibitors CDKN2A and CDKN2C). Seven genes were engaged in G2/M transition with the majority negatively regulating the transition (cyclin CCNB2 for promotion, negative regulator WEE1 and YWHAQ, genes associated with p53 signaling including MDM2 and CHEK1, stress sensors GADD45B and GADD45G). Twelve genes participated in the metaphase to anaphase transition (anaphase promoting complex and the associated molecules: ANAPC4, 10, 11, CDC16, 20, 23, 27, PTTG1/securin, FZR1/Cdh1; spindle checkpoint related genes: MAD2L1/MAD2, BUB1, and TTK/Mps1).

**Figure 5:**
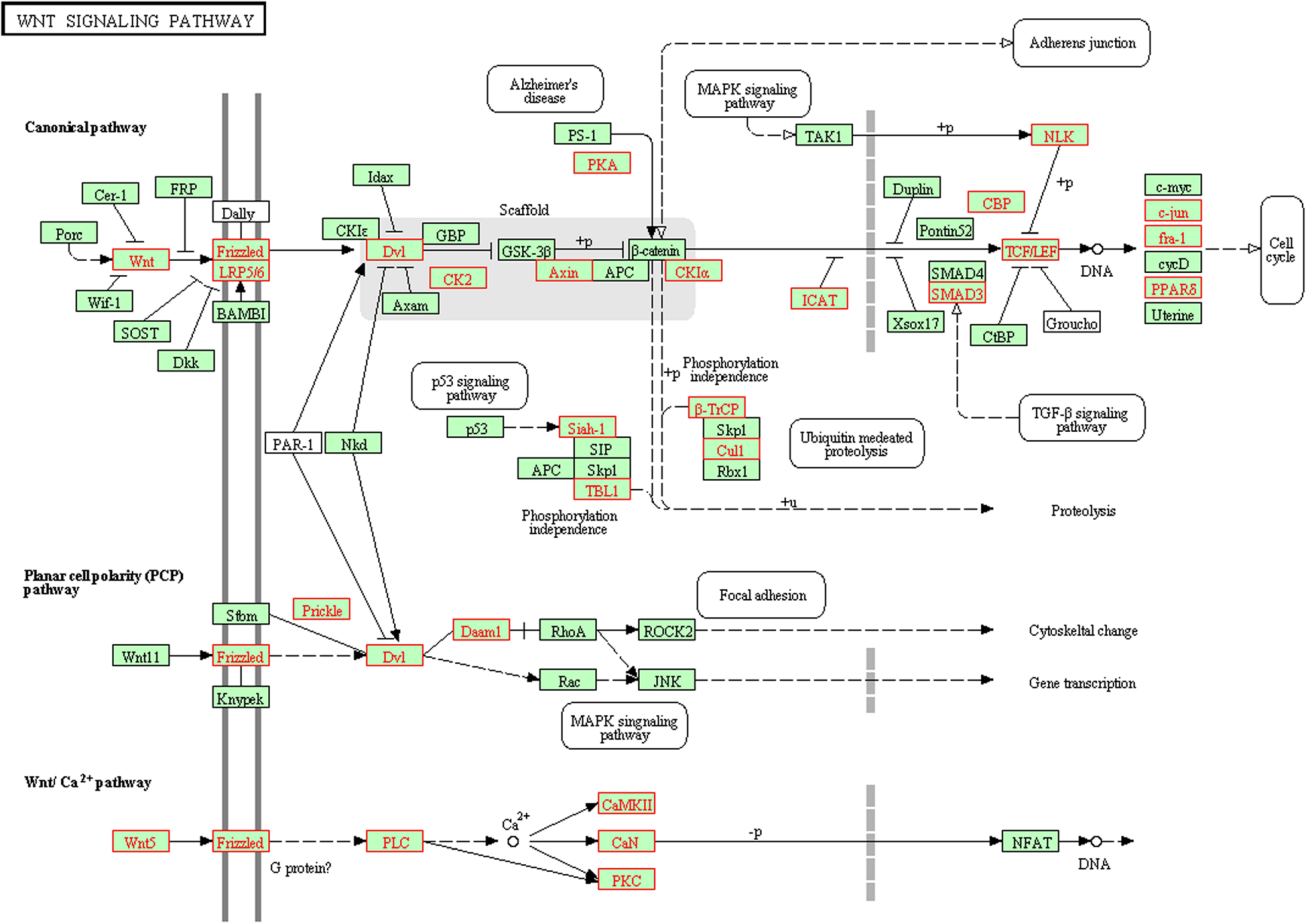
**Coordinately up-regulated genes of WNT signaling pathway in HCV versus HIV.** The pathway figure is adapted from Kyoto Encyclopedia of Genes and Genomes (KEGG; http://www.genome.jp/kegg/). The proteins encoded by the coordinately up-regulated genes in the HCV group are highlighted in red.

For WNT signaling pathway, 36 out of 148 gene members were coordinately up-regulated in HCV (Table 2; Figure 5). In addition to the genes encoding for receptors and ligands (FZD2, FZD7, LRP5, WNT10A, WNT5B, and WNT7A), the majority of the core genes populated the arms of β-catenin signaling and the WNT Ca^2+^ signaling. Nine genes were associated with the activation of β-catenin-dependent transcription and the expression of the targeted genes (DVL2, CSNK2A2/CK2, PRKACG/PKA, EP300/CBP, SMAD3, LEF1, TCF7, PPARD, FOSL1/fra-1, JUN), whereas 8 genes were involved in the negative regulation of this process (β-catenin phosphorylation and degradation complexes: AXIN2 and CSNK1A1L/CKIα, CUL1, FBXW11/β-TrCP, SIAH1, TBL1XR1/TBL1; kinases: CTNNBIP1/ICAT and NLK). Along the arm of WNT Ca^2+^ signaling, 9 core genes covering all the molecules of this branch were detected.

### Significant alteration of cancer-related signaling pathways in HIV/HCV co-infection

Both p53 signaling (FDR = 0.029) and renal cell carcinoma pathways (FDR = 0.036) were significantly down-regulated in HH versus HIV and HCV, respectively. Twenty-six out of 67 gene members of the p53 pathway were coordinately down-regulated in the HH group (Table 2; Additional file 6), which included 4 genes interacting with p53 (CHEK1, MDM2, MDM4, and CDKN2A/p14arf), 8 genes encoding for the arm of cell cycle arrest (cyclins: CCNB2, CCND1, CCNE1, CCNE2; Cyclin-CDK inhibitor CDKN1A/p21; stress sensors: GADD45A and GADD45B), and 7 genes involved in apoptosis (BID, CASP8, CASP9, EI24/PIGs, TP53I3/PIGs, PMAIP1/Noxa, FAS). The remaining core genes spread along the branches of inhibition of angiogenesis (SERPINE1/PAI, CD82/KAI, THBS1/TSP1), DNA repair and damage prevention (SESN1, 2, 3), and p53 negative feedback (CCNG2 and RCHY1). It was also noted that there was a trend in the down-regulation of p53 signaling in HCV versus HIV (FDR = 0.12), which was contributed by 25 core genes. The spread of these core genes in p53 pathway displayed a similar pattern to that observed in the comparison between HIV and HH (see Additional file 6 for common and distinct core genes detected in these 2 comparisons).

As for renal cell carcinoma pathway, 18 out of 70 gene members of the pathway were coordinately down-regulated in the HH group (Table 2; Additional file 7), which were featured by the genes encoding for several growth factors (TGFB2, TGFB3, VEGFA, PDGFB) and genes involved in cell survival and proliferation (GRB2-KRAS/NRAS-RAF1-MAPK3/ERK-JUN/AP1 leading to cell cycle progression and PIK3CA/PI3K leading to cell survival).

### qPCR confirmation of altered gene expression detected by microarray

To further confirm the altered gene expression detected by microarray analysis, mRNA expression levels of the selected DEGs/core genes were measured by quantitative PCR (qPCR; Table 3). The genes were selected based on the coverage of different levels and directions of fold change, different group comparisons, and/or biological significance. The fold changes for each pairwise comparison evaluated by qPCR were consistent with the results obtained from microarray, which confirmed the reliability of our microarray data.

**Table 3:** **qPCR confirmation of altered gene expression detected by microarray**

**Gene symbol**	**KEGG pathways the validated genes involved**	**Group comparison**	**Fold change by microarray**	**Fold change by qPCR**
IL6	Cytokine- cytokine receptor interaction; HIF-1 signaling; FOXO signaling; PI3K-Akt signaling; Toll-like receptor signaling	HIVvsHH	5.1	4.8
IL6		HIVvsCTR	4.6	3.5
CXCR4	Cytokine-cytokine receptor interaction; Chemokine signaling; Endocytosis; Axon guidance; Leukocyte transendothelial migration	HCVvsCTR	−1.5	−2.5
TAOK1	MAPK signaling pathway	HCVvsHH	−2.0	−3.7
TAOK1		HIVvsHH	−2.2	−2.3
EGR2	Hepatitis B; HTLV-I infection; Viral carcinogenesis	HCVvsHH	8.8	10.2
EGR2		HIVvsHH	8.8	9.3
PPP3CB	MAPK signaling; WNT signaling; Apoptosis; Calcium signaling	HHvsCTR	1.8	1.4
WNT7A	WNT signaling; Hedgehog signaling; Hippo signaling; HTLV-I infection	HCVvsHH	2.1	2.6
DUSP2	MAPK signaling pathway	HCVvsHH	2.6	2.8
DUSP2		HIVvsHH	2.1	2.7
DUSP2		HCVvsCTR	2.5	3.2
MCM2	DNA replication; Cell cycle	HCVvsHH	−1.5	−2.9
MCM2		HIVvsHCV	1.5	1.9
*GZMB*	NK cell mediated cytotoxicity; Regulation of actin cytoskeleton; Transcriptional misregulation in cancer; Autoimmune thyroid disease	HCVvsHH	−2.4	−3.1
*GZMB*		HIVvsHH	−1.5	−2.0
*GZMB*		HIVvsHCV	1.6	1.6
*KLRD1*	NK cell mediated cytotoxicity pathway; Antigen processing and presentation; Graft-versus-host disease	HCVvsHH	−2.2	−2.9
*KLRD1*		HIVvsHH	−1.3	−1.5
*KLRD1*		HIVvsHCV	1.6	2.0
*WEE1*	Cell cycle	HIVvsHCV	1.3	1.9
*SESN1*	p53 signaling	HIVvsHH	1.6	2.1

Fold change by qPCR was obtained from the mean expressions of the tested genes in each group. All values represent fold changes between expression levels of the first group versus expression levels of the second group. Minus sign indicates down-regulation in the first group whereas positive sign indicates up-regulation in the first group. Housekeeping gene GAPDH was used as an internal control and the normalizer in qPCR.

Genes in Italic font indicate the core genes detected by GSEA.

## Discussion

Our study has investigated the transcriptome distinctions of PBMCs from HIV, HCV mono- and co-infected patients (Additional file 1). Pairwise comparisons (HCV versus HH, HIV versus HH, and HIV versus HCV) were carried out and differentially expressed genes as well as significantly altered pathways were identified (Table 1; Additional files 3 and 4). Across the DEG lists, immune system and metabolic processes were the major biological themes derived from the DEGs. At the pathway level, GSEA identified a panel of significantly altered pathways for each pairwise comparison, which could be divided into the following five biological categories: (1) immune system; (2) signaling transduction and cell cycle; (3) metabolism; (4) genetic information processing; and (5) human diseases (Additional file 4). As GSEA enabled a more comprehensive detection of genes contributing to the enrichment of the pathways correlated with specific patient groups, the subsequent discussion will focus on the pathways significantly associated with each infection group revealed by GSEA. Since the first three categories had the most direct relevance to HIV disease and HCV-mediated carcinogenesis, our subsequent discussion shall center on the crucial pathways from these categories, including cytotoxicity, cytokine-cytokine receptor interaction, cell cycle, WNT, and p53 signaling pathways (Table 2).

Under the category of immune system, the most unique finding in our study is the significant up-regulation of cytotoxicity pathway in the former versus the latter group across the 3 pairwise comparisons (HH versus HIV, HH versus HCV, HIV versus HCV; FDR from <0.001 to 0.019; Figure 3; Table 2). Our study is the first to report the significant alterations of this pathway differentiating between HIV, HCV mono- and co-infection, which is also supported by previous findings in HIV/HCV co-infection [30,31,33,34]. Recently, two PBMC studies have implicated this pathway. One of them reported its enrichment in the significantly up-regulated genes in co-infected patients versus controls [34], and the other showed NK cell signaling in the expanded network constructed from the gene signature in HH versus HCV group [30]. In addition, the up-regulation of cytotoxicity pathway in HH versus HIV as well as in HIV versus HCV has been found in CD4+ T cells [31], and high percentages of cytotoxic CD8+ T cells associated with liver fibrosis in co-infected patients have been detected by the flow cytometry study [33].

Notably, a substantial portion of the core genes contributing to the significant regulation of cytotoxicity pathway encoded for killer-cell immunoglobulin-like receptors (KIRs) (Table 2). Among the 8 core genes present across 3 pairwise comparisons, 4 genes encoded for KIRs (KIR2DL1, KIR2DL3, KIR2DL5A, and KIR3DL2), which may implicate that the KIRs, with their crucial roles in virus infection, were under such tight regulations that their expression levels were able to reflect specific patterns of particular virus-host interactions. This speculation was in line with the previous study in which it was found that elevated frequencies of NK cells expressing HLA-C binding KIRs, such as the aforementioned KIR2DL1 and KIR2DL3, were associated with HCV clearance [35]. Moreover, the KIR2DL3: HLA-C1 homozygosity had the protective effects in HCV-infected individuals exposed to low inocula [36], possibly due to their lower avidity interactions that resulted in an advantageous activation of NK cell against HCV. The similar mechanism has also been proposed for the observation that resistance to HIV infection was associated with KIR3DL1 homozygosity in the absence of its ligand HLA-Bw4 in an African cohort [37]. Interestingly, KIR3DL1 was also detected as one of the coordinately up-regulated core genes in HH versus either HIV or HCV in this study (Figure 3). Besides KIRs, 5 up-regulated core genes encoding for activation receptors were also present in HH versus either HCV or HIV, of which NCR3/NKp30 was noteworthy. The increased expression of NKp30 was protective against HCV infection in high-risk individuals and inhibited HCV replication *in vitro* [38]. In HIV/HCV co-infected individuals, a significantly higher proportion of NK cells expressing NKp30 on their surface were detected compared to the HIV group [29]. Given the fact that most of the aforementioned core genes played protective roles against virus infection and the cytotoxicity function was impaired in HIV and HCV infection [39-41], the up-regulation of the cytotoxicity pathway in co-infection would be most likely to reflect a compensatory effect of the cytotoxic cells imposed by aberrant activation or anergy arising from the co-infection.

Another prominent change observed in the immune system was the down-regulation of cytokine-cytokine receptor interaction pathway in HH versus either HCV or HIV (Table 2; Figure 4). For the core genes commonly down-regulated in HH in both comparisons, some of them were previously reported with decreased expression in HH versus HCV, such as IL8 and TNF-α [25,26,28]. For the core genes uniquely detected in one of the two comparisons, the coordinate down-regulation of IFNG, IFNGR2 and FAS was pronounced only in HH versus HIV, whereas the coordinate down-regulation of CX3CL1, interferon-α genes (IFNA4, 8, 10 and IFNAR2) and TGF-β genes (TGFB2, 3, and TGFBR2) was observed only in HH versus HCV. While the exact mechanisms underlying these distinct immune activation profiles are not well understood, previous studies have suggested some crucial roles of these genes, such as the elevation of CX3CL1 as a marker of liver fibrosis/injury [29], TGF-β as a potent inducer of fibrogenesis [26], and interferon-α inhibiting HCV replication [42]. Interestingly, a recent study has reported that an effective interferon-γ mediated inhibition of HCV replication by NK cells is associated with spontaneous clearance of HCV in HIV+ patients [43], which is in line with our simultaneous detections of both the dysregulation of NK cytotoxicity pathway and the decreased expression of IFNG in co-infected individuals.

For signaling transduction and cell cycle pathways, the respective up- and down-regulation of cell cycle and WNT signaling pathways in HIV versus HCV were noted, which may reflect the differential impact of these two viruses on cell proliferation. While the genes involved in the promotion of G1/S transition and the induction of arrest in G2/M transition were up-regulated in the HIV group (Additional file 5), the activation of cell cycle through WNT/β-catenin was more pronounced in the HCV group (Figure 5). Consistently, the differential regulation of cell cycle between HIV and HCV was also prominent in microarray studies on CD8+ and CD4+ T cells, and the similar pattern of up-regulation of genes involved in promotion of G1/S transition along with G2 arrest was also detected in CD4+ T cells in HIV versus HCV [31,32]. Taken together, these observations could possibly suggest that HCV-infected cells may experience cell cycle dysregulation at a less severe level than HIV-infected cells. In support of this speculation, a recent study has shown that T cells in HIV/HCV co-infected patients were destroyed at a slower rate than in HIV mono-infected patients [44]. In addition, previous studies have demonstrated the oncogenic activity of HCV core protein, which induces cell proliferation through various processes such as activating RB/E2F signaling [45,46]. However, there were also some discrepant results [47,48], which could be attributed to the variations in the cohort and different cell systems (*in vivo* versus *in vitro*).

As for the oncogenic activity of HCV proteins, it was not only evident in the up-regulation of WNT pathway in HCV versus HIV, but also manifested by the down-regulation of p53 signaling pathway in HH versus HIV. For WNT signaling pathway, the up-regulation of FZD7 receptors associated with the activation of the WNT/beta-catenin pathway, which was observed in this study in HCV versus HIV (Figure 5), was a common molecular event in HCC [49]. The up-regulation of PPARD (peroxisome proliferator-activated receptor, a central regulator of triglyceride homeostasis) in WNT/beta-catenin pathway was also detected in this study. The persistent activation of PPAR was essential for the pathogenesis of hepatic steatosis and HCC induced by HCV infection in the animal model [50]. For p53 signaling pathway, the genes responsible for cell cycle arrest, apoptosis, inhibition of angiogenesis, DNA repair and damage prevention, were all coordinately down-regulated in HH versus HIV (Additional file 6), which may contribute to HCV-induced carcinogenesis. Interestingly, there was also a trend in the down-regulation of p53 pathway in HCV versus HIV (FDR = 0.12), and the core genes detected in this comparison displayed a similar pattern to that observed in the comparison between HIV and HH (Additional file 6). Hence, it is possible that this gene expression signature may reflect a specific pattern directly related to the oncogenic activity of HCV proteins. These observations were also consistent with previous studies showing that p53 effector function could be compromised by the functional inactivation of tumor suppressor protein promyelocytic leukemia through the HCV core protein in HCV-infected cells [51], and that the NS3 protein could form complexes with p53 and repress its function [52].

Among the pathways of human diseases, the oncogenic role of HCV was further highlighted by the up-regulation of renal cell carcinoma pathway in HCV versus HH (Additional file 7). Notable was the up-regulation of HIF-α (EPAS1 and HIF1A), which led to the up-regulation of growth factors (TGFB2, TGFB3, VEGFA, PDGFB) and glucose transporter SLC2A1/GLUT1. Previous studies have shown that the expression of HCV protein activates HIF-1 by normoxic stabilization of its α subunit (HIF-α), resulting in increased expression of HIF-controlled genes, many of which are involved in tumor growth and metastasis, such as VEGF and TGF-β [53-55].

It was also noted that metabolic pathways, particularly those involved in energy metabolism such as oxidative phosphorylation (OXPHOS) and TCA cycle, were consistently up-regulated in HH versus either HIV or HCV (Additional file 4). Similarly, gene sets including carbohydrate, lipid, amino acid, and nucleotide metabolism were also up-regulated in the co-infection group in CD4+ T cells [31]. This up-regulation in HH may implicate a compensatory event at a higher level than in mono-infection, possibly arising from more insufficient energy supply due to the more severe OXPHOS impairment imposed by both HIV and HCV [55,56]. For genetic information processing, all the pathways were up-regulated in HH versus either HIV or HCV, which involved processes at the DNA level including recombination, repair, and replication, and at translational or posttranslational level including tRNA synthesis and proteasome (Additional file 4), implying the more severely perturbed human gene machinery in HIV/HCV co-infection.

A few limitations of this study should be noted. First, our data from PBMCs only provided the overall changes of the transcriptomes in circulating mononuclear cells. A more thorough understanding of the mechanisms underlying these changes would require further transcriptome profiling at the individual cell type level, as we previously did on monocytes and T cell subsets [56-58]. Secondly, although our results pinned down to important KIR genes such as KIR2DL3 and KIR3DL1, the genotype of HLA was not available and the sample size also limited further analysis of genotype association. Thirdly, this study used a relatively small sample size appropriate for the pilot investigation. Future studies using larger sample size are thus warranted to confirm the results as well as to explore the role of KIRs and HLA genotype in co-infection. Finally, although the GSEA identified a panel of significantly altered pathways differentiating between HIV, HCV mono- and co-infection, the predictive nature of GSEA as the common limitation of statistical tools should be noted. Future biological experiments focusing on the clinically and pathologically relevant pathways shall be carried out, which can not only further validate the novel use of PBMCs, but also provide more incisive mechanistic insights in HIV/HCV co-infection.

## Methods

### Study subjects

Blood samples (8–10 ml in EDTA) were obtained by venipuncture from 27 subjects belonging to the following clinical groups: HCV infected patients (*n* = 5), HIV infected patients (*n* = 7), HIV/HCV co-infected patients (*n* = 10), and HIV/HCV sero-negative healthy controls (*n* = 5; Additional file 1). The co-infected patients were from Hospital Carlos, Madrid, Spain, and the rest were from Westmead Hospital, Sydney. Institutional Ethics Committee of the Hospital Carlos, Madrid, Spain, independently approved the use of the co-infected samples, and all the other blood samples were collected after individual informed written consent using a protocol approved by the Sydney West Area Health Services Research Ethics Committee.

### Isolation of PBMCs and RNA extraction

PBMCs were extracted immediately after the collection of whole blood using Ficoll-hypaque density gradient centrifugation [59]. Total RNA was isolated from purified cells using RNeasy Mini kit (Qiagen Pty Ltd., Clifton Hill, Victoria, Australia) with an integrated step of on-column DNase treatment.

### cRNA preparation, microarray hybridization and scanning

RNA quality was checked by Agilent Bioanalyzer and RNA Integrity Scores were higher than 7 for all the samples. cRNA amplification and labeling with biotin were performed using Illumina TotalPrep RNA amplification kit (Ambion, Inc., Austin, USA) with 250 ng total RNA as input material. cRNA yields were quantified with Agilent Bioanalyzer and 750 ng cRNAs were hybridized to Illumina HumanHT-12 v3 Expression BeadChips (Illumina, Inc., San Diego, USA). Each chip contained 12 arrays and each array contained >48,000 gene transcripts, of which, 46,000 were derived from human genes in the National Center for Biotechnology Information (NCBI) Reference Sequence (RefSeq) and UniGene databases. All reagents and equipment used for hybridization were purchased from Illumina, Inc. According to the manufacturer’s protocol, cRNAs were hybridized to arrays for 16 hours at 58°C before being washed and stained with streptavidin-Cy3. Then the beadchips were centrifuged to dry and scanned on the Illumina BeadArray Reader confocal scanner. To minimize the batch effect, the microarray chips were all processed at the single site using the same platform with the identical setting of the parameters by the same experimenter.

### Analysis of differentially expressed genes

The quality of the entire data set was assessed by box plot and density plot of bead intensities, density plot of coefficient of variance, pairwise MAplot, pairwise plot with microarray correlation, cluster dendrogram, and non-metric multidimensional scaling using R/Bioconductor and the lumi package [60]. Based on the quality assessment, all 27 samples were deemed suitable for further analysis. Data normalization was performed using log2 transform and a robust spline normalization (RSN) implemented in the lumi package for R/Bioconductor [60,61]. To reduce false positives of differentially expressed genes, genes below detectable limit (based on a detection p value cut-off 0.01) were removed from the dataset. A linear model fit in conjunction with an empirical Bayes statistics was used to identify candidate DEGs [62]. P values were corrected for multiple testing using FDR adjustment. Each patient group was first compared to the control group and we then focused on pairwise comparisons between the 3 patients groups. Candidate DEGs with fold change >1.5 and FDR < 0.05 were identified for each of the comparisons. The heatmap including all the study subjects was produced by heatmap.2 function of the gplots package from R/Bioconductor. The lists of the DEGs derived from the pairwise comparisons between the patients groups were submitted to PANTHER classification system for categorizing the DEGs based on biological processes defined by GO terms [63,64].

### Gene set enrichment analysis

GSEA was used for the investigation of global dysregulations of the biological pathways between the 3 patients groups. The gene sets were from MsigDB [65], catalog C2 functional sets, subcatalog KEGG pathways, which included 186 gene sets from pathway databases. For each group comparison, GSEA was performed using the normalized data of the entire 48,803 transcripts (GSEA version 2.0.14, Broad Institute http://www.broad.mit.edu/gsea). First, a ranked list was obtained by ranking all genes according to the correlation between their expression and the group distinction using the metric signal to noise ratio. Then the association between a given gene set and the group was measured by the non-parametric running sum statistic termed the enrichment score (ES), which was calculated by walking down the ranked list (increasing ES when encountering a gene in the given gene set and decreasing ES when encountering a gene not in the gene set). To estimate the statistical significance of the ES, a nominal p value was calculated by permuting the genes 1,000 times. To adjust for multiple hypothesis testing, the maximum ES was normalized to account for the gene set size (NES) and the FDR corresponding to each NES was also calculated. Along with the pathway enrichment results, the details report for each significant pathway was simultaneously generated. This report has listed the details of each gene member in columns, one of which indicates whether this gene member is “core enrichment gene” or not. The core enrichment genes account for the enrichment signal of the pathway and the inspection of them can reveal a biologically important subset within the pathway [65].

### Real-time quantitative PCR

Twelve genes were selected for validation based on the coverage of different levels and directions of fold change, different group comparisons, and/or biological significance. Purified total cellular RNA was used for reverse transcription with oligo d(T) and Superscript III followed by RNase H treatment (Invitrogen Life Technologies). The cDNA was then subjected to qPCR in a 96-well format in triplicate reactions with defined primers and SYBR Green (Invitrogen Life Technologies). The qPCR reactions were carried out using Mx3005P™ QPCR System (Stratagene). The mean expressions of the tested genes in each group were obtained and the housekeeping gene GAPDH was used as an internal control and the normalizer for all data. The fold change was calculated by the relative quantitation method 2^-(ddCt)^. Primer sequences for each transcript are available from the authors upon request.

## Conclusions

Our study is the first to identify the differential regulation of cytotoxicity pathway discriminating between HIV, HCV mono- and co-infection, which may reflect the distinct patterns of virus-host cell interactions underlying disease progression. Further inspection of cytotoxicity pathway has pinned down to the expression of the KIR genes to be associated with specific patterns of particular virus-host interactions. Moreover, the down-regulation of cytokine-cytokine receptor interaction and the up-regulation of metabolic pathways in HH versus HIV or HCV reflected aberrant immune activation and more severely perturbed metabolism in the co-infection. Between HIV and HCV, the differential regulation of cell cycle and WNT signaling pathways may suggest the distinct impact of HIV and HCV on cell proliferation and differentiation. In addition, the up-regulation of WNT and retinal carcinoma pathways in HCV (versus HIV and HH, respectively) may indicate the oncogenic role of this virus. This oncogenic role was further manifested by the down-regulation of the components of p53 pathway in HH versus HIV, consisting of inhibition of angiogenesis as well as DNA repair and damage prevention. Future studies on the regulation of these pathways and the corresponding core enrichment genes may provide a detailed understanding of the molecular mechanisms involved, which may also aid the development of therapeutic interventions.

## Additional files

Additional file 1:
**Clinical profiles of study subjects.** The table listing the clinical profiles of study subjects. Additional file 2:
**Differentially expressed genes derived from the comparisons of HH, HIV, or HCV versus CTR.** The list of differentially expressed genes for the comparisons of HH, HIV, or HCV versus CTR. Additional file 3:
**Differentially expressed genes derived from the pairwise comparisons between HIV, HCV, and HH.** The list of differentially expressed genes for the comparisons of HIV versus HH, HCV versus HH, and HIV versus HCV. Additional file 4:
**Differentially regulated pathways from GSEA for the pairwise comparisons between HIV, HCV, and HH.** The list of significant pathways for the comparisons of HIV versus HH, HCV versus HH, and HIV versus HCV. Additional file 5:
**The coordinately up-regulated cell cycle pathway in HIV versus HCV.** The pathway figure is adapted from Kyoto Encyclopedia of Genes and Genomes (KEGG; http://www.genome.jp/kegg/). The proteins encoded by the coordinately up-regulated genes in the HIV group are highlighted in red. Additional file 6:
**The coordinately down-regulated p53 signaling pathway in HH versus HIV (FDR=0.029) and in HCV versus HIV (a trend of down-regulation with FDR=0.12).** The pathway figure is adapted from Kyoto Encyclopedia of Genes and Genomes (KEGG; http://www.genome.jp/kegg/). The red and blue front colors highlight the proteins encoded by the coordinately down-regulated genes in HH versus HIV and in HCV versus HIV, respectively. The background color filled with pink highlights the proteins encoded by the commonly down-regulated genes found in both comparisons (HH versus HIV and HCV versus HIV). Additional file 7:
**The coordinately up-regulated renal cell carcinoma pathway in HCV versus HH.** The pathway figure is adapted from Kyoto Encyclopedia of Genes and Genomes (KEGG; http://www.genome.jp/kegg/). The proteins encoded by the coordinately up-regulated genes in the HCV group are highlighted in red.
