# Supplementary figures and images for: Differential regulation of cytotoxicity pathway discriminating between HIV, HCV mono- and co-infection identified by transcriptome profiling of PBMCs
Source: Virol J. 2015 Jan 27;12:4. doi: 10.1186/s12985-014-0236-6 (PMC4312599; doi:10.1186/s12985-014-0236-6)

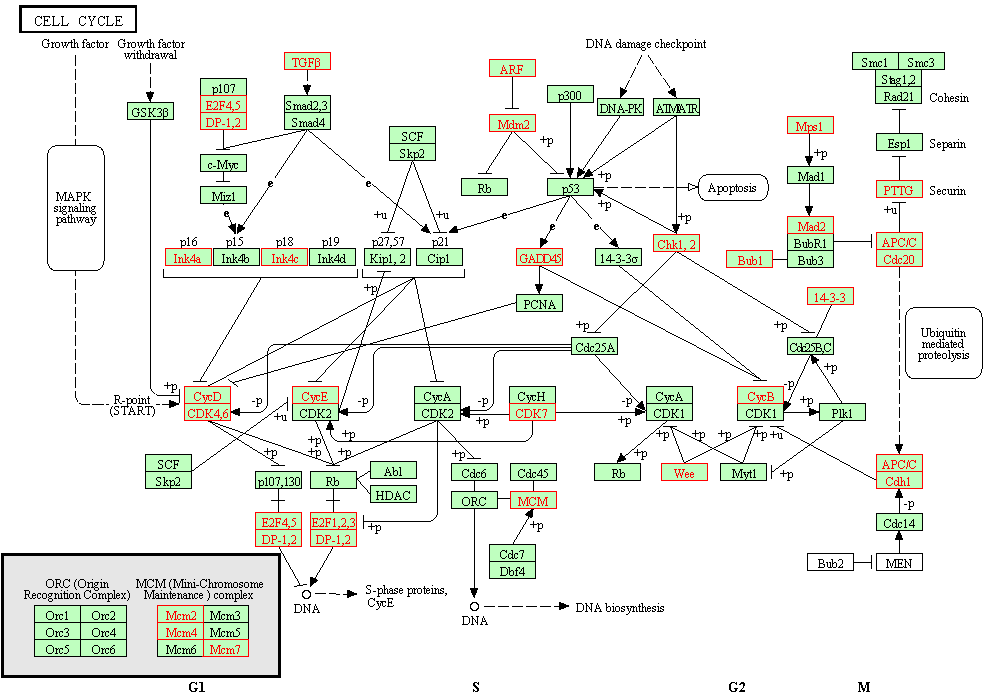

Supplement: Additional file 5: — The coordinately up-regulated cell cycle pathway in HIV versus HCV. The pathway figure is adapted from Kyoto Encyclopedia of Genes and Genomes (KEGG; http://www.genome.jp/kegg/). The proteins encoded by the coordinately up-regulated genes in the HIV group are highlighted in red. [file 12985_2014_236_MOESM5_ESM.tiff]

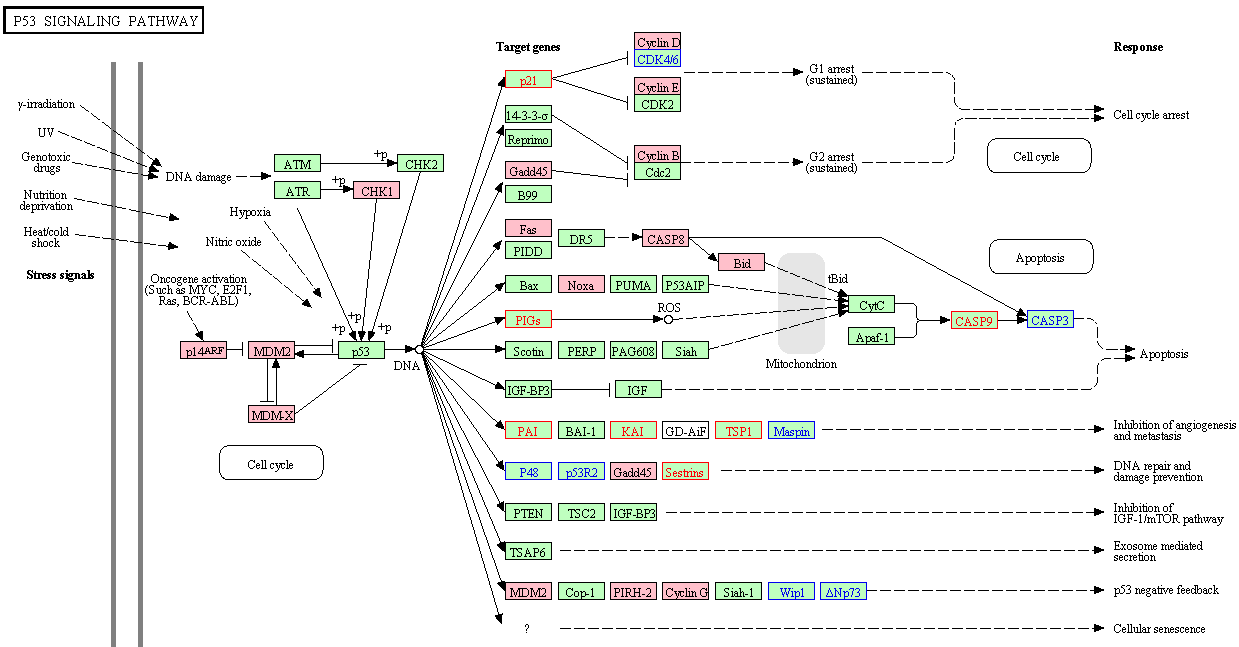

Supplement: Additional file 6: — The coordinately down-regulated p53 signaling pathway in HH versus HIV (FDR=0.029) and in HCV versus HIV (a trend of down-regulation with FDR=0.12). The pathway figure is adapted from Kyoto Encyclopedia of Genes and Genomes (KEGG; http://www.genome.jp/kegg/). The red and blue front colors highlight the proteins encoded by the coordinately down-regulated genes in HH versus HIV and in HCV versus HIV, respectively. The background color filled with pink highlights the proteins encoded by the commonly down-regulated genes found in both comparisons (HH versus HIV and HCV versus HIV). [file 12985_2014_236_MOESM6_ESM.tiff]

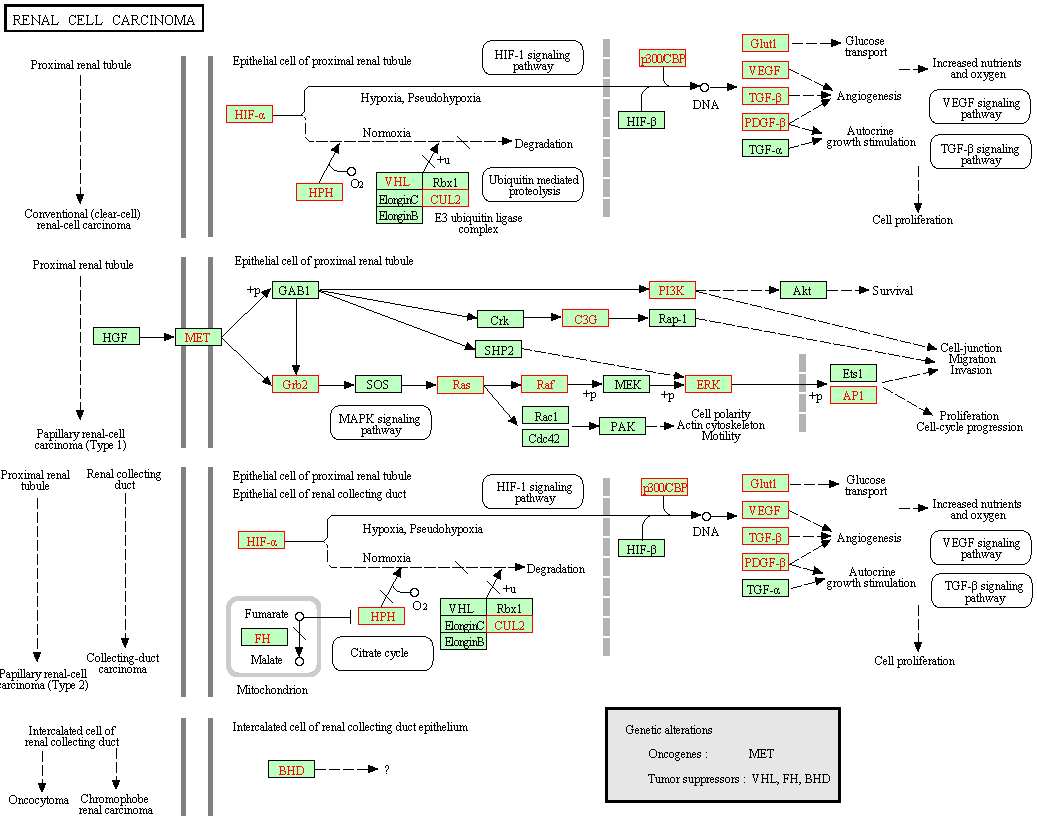

Supplement: Additional file 7: — The coordinately up-regulated renal cell carcinoma pathway in HCV versus HH. The pathway figure is adapted from Kyoto Encyclopedia of Genes and Genomes (KEGG; http://www.genome.jp/kegg/). The proteins encoded by the coordinately up-regulated genes in the HCV group are highlighted in red. [file 12985_2014_236_MOESM7_ESM.tiff]
